# Freezing method for rock cross-cut coal uncovering I: Mechanical properties of a frozen coal seam for preventing outburst

**DOI:** 10.1038/s41598-019-52879-y

**Published:** 2019-11-08

**Authors:** Jiwei Yue, Gaowei Yue, Zhaofeng Wang, Minmin Li, Binbin Wang, Fenghua An

**Affiliations:** 1 School of Safety Science and Engineering, Henan Polytechnic University, 2001# Century Avenue, Jiaozuo, 454000 P.R. China; 2 School of Civil Engineering, Henan Polytechnic University, 2001# Century Avenue, Jiaozuo, 454000 P.R. China

**Keywords:** Civil engineering, Natural hazards, Geophysics, Tectonics

## Abstract

A comprehensive technology is proposed to realize fast and safe rock cross-cut coal uncovering (RCCCU) based on artificial freezing engineering method. This comprehensive technology includes four steps, namely, drilling a borehole, wetting the coal body by water injection, gas drainage and freezing the coal seam by liquid nitrogen injection. In this paper, the compressive strength, tensile strength and shear strength of frozen coal specimens are tested to obtain the mechanical parameters of the specimen. Then, for RCCCU under freezing temperatures, the outburst prevention effects are calculated and quantitatively analysed with regard to three aspects, namely, the enhancement of coal the mechanical properties, the reduction in the coefficient of outburst hazard (COH) in the distressed zone and the reduction in the interfacial elastic energy ratio (IEER) between the coal seam and the roof/floor. The results show that a considerable improvement in the mechanical properties of frozen coal and that the coal mechanical parameters, such as the compressive strength and the tensile strength, increase linearly with decreasing temperature. The coefficient of outburst hazard in the distressed zone decreases rapidly and drops from above 0.8 to below 0.3. The interfacial elastic energy ratio is greatly reduced from dozens of times of that of the roof/floor before freezing to several times of that of the roof/floor after freezing, which effectively weakens the sudden change of the elastic energy at the coal-rock interface.

## Introduction

Coal and gas outburst is one of the common disasters in underground production in coal mines. Statistics shows that, the working face of the roadway has the most outbursts, followed by the stope face, whereas rock cross-cut coal uncovering (RCCCU) has the fewest outbursts. Rock cross-cut coal uncovering is associated with relatively few outbursts but has the highest proportion of outbursts with the greatest average strength, which is more than 6 times that of other outbursts, and more than 80% of the super-large outbursts occur in the rock cross-cut coal uncovering working face^1–5^. The frequency of outburst accidents and high outburst strength in rock cross-cut coal uncovering are attributed to the stress state of the uncovered coal seam and rock in front of the working face, which are prone to sudden changes, resulting in a large release of elastic potential energy from the coal seam and gas expansion energy^6–8^.

Coal strength is the key factor in resisting outbursts and determines whether the coal will be destroyed under the *in-situ* stress and gas pressure. Currently, the main measures for improving coal strength are grouting reinforcement and the use of a metal framework, which have obtained some good results in preventing outburst in rock cross-cut coal uncovering^9,10^. However, significant limitations are associated with these measures, e.g., it is difficult for grouting material to diffuse in coal seams, and the range of reinforcement is limited^11^. Furthermore, the gas pressure and gas content in coal seams gradually increase with the mining depth, *in-situ* stress and ground temperature. Thus, the risk of coal and gas outburst also increases. The danger of coal and gas outbursts are difficult to eliminate rapidly and effectively using current measures, which include gas pre-drainage and hydraulic flushing^12,13^. Traditional technology with a focus on safety requires a long engineering period in rock cross-cut coal uncovering. Therefore, it is of considerable theoretical and practical value to develop a new technology for safe and rapid rock cross-cut coal uncovering.

Liquid nitrogen freezing is a mature technique in the artificial frozen soil field. Liquid nitrogen gasifies and adsorbs heat in a frozen tube to realize soil freezing, improve soil strength and facilitate the next step in construction. Liquid nitrogen freezing has better characteristics than brine circulation freezing, including a lower freezing temperature, a higher frozen soil strength and a faster freezing speed. Liquid nitrogen freezing technology has been widely used in foundation reinforcement in urban underground engineering, the tunnelling of railways and highways and in mine construction^14–21^.

There have been a significant number of experimental reports on the considerable high strength of a frozen coal body because the coal body contains a certain amount of water^22–24^. At the same time, the gas adsorption capacity in coal increases with decreasing temperature, which results in a lower gas pressure in a confined space^25–28^. Both of these results are extremely beneficial for safe coal uncovering. Therefore, based on artificial freezing engineering methods, liquid nitrogen freezing and its ancillary technology (such as drilling boreholes and wetting the coal body by water injection) can be applied to rock cross-cut coal uncovering, which could effectively eliminate the risk of coal and gas outbursts. Using the conventional method in the process of rock cross-cut coal uncovering can last for more than ten months, especially for soft coal seams, which costs hundreds of thousands of dollars a month. A novel method is proposed for rock cross-cut coal uncovering that can be implemented in approximately 2 months, and the coal mines can be mined in advance, resulting in extremely large indirect economic benefits.

In this paper, tests are performed to determine the physico-mechanical properties and stress state of the coal-rock mass during the process of rock cross-cut coal uncovering and the compressive strength, tensile strength and shear strength of frozen coal. The mechanical strength of coal, the risk coefficient of coal and gas outburst and the contrast coefficient for the elastic energy between coal and the roof/floor are calculated and analysed to prove the effectiveness of eliminating coal and gas outbursts in rock cross-cut coal uncovering using the freezing method.

## Basic Theory of Outbursts In Coal Seams

### Coefficient of outburst hazard in distressed zone

1. Width of distressed zone
  In rock cross-cut coal uncovering (as shown in Fig. 1(a)), the stress balance in the original stratum is destroyed by the drivage roadway, and the stress is redistributed in the coal. Usually, a distressed zone and a stress concentration zone form in front of the excavation space (Fig. 1(b)). To achieve the stress concentration effect, the coal at the edge is first crushed, which significantly reduce the coal strength, and the distressed zone can only withstand low stresses. The width of the distressed zone and its withstanding capacity significantly affect coal and gas outbursts. The distressed zone act as a protective wall: the thinner the wall, the larger the gas pressure gradient and the higher possibility that the wall will be damaged in an outburst.

**Figure 1:**
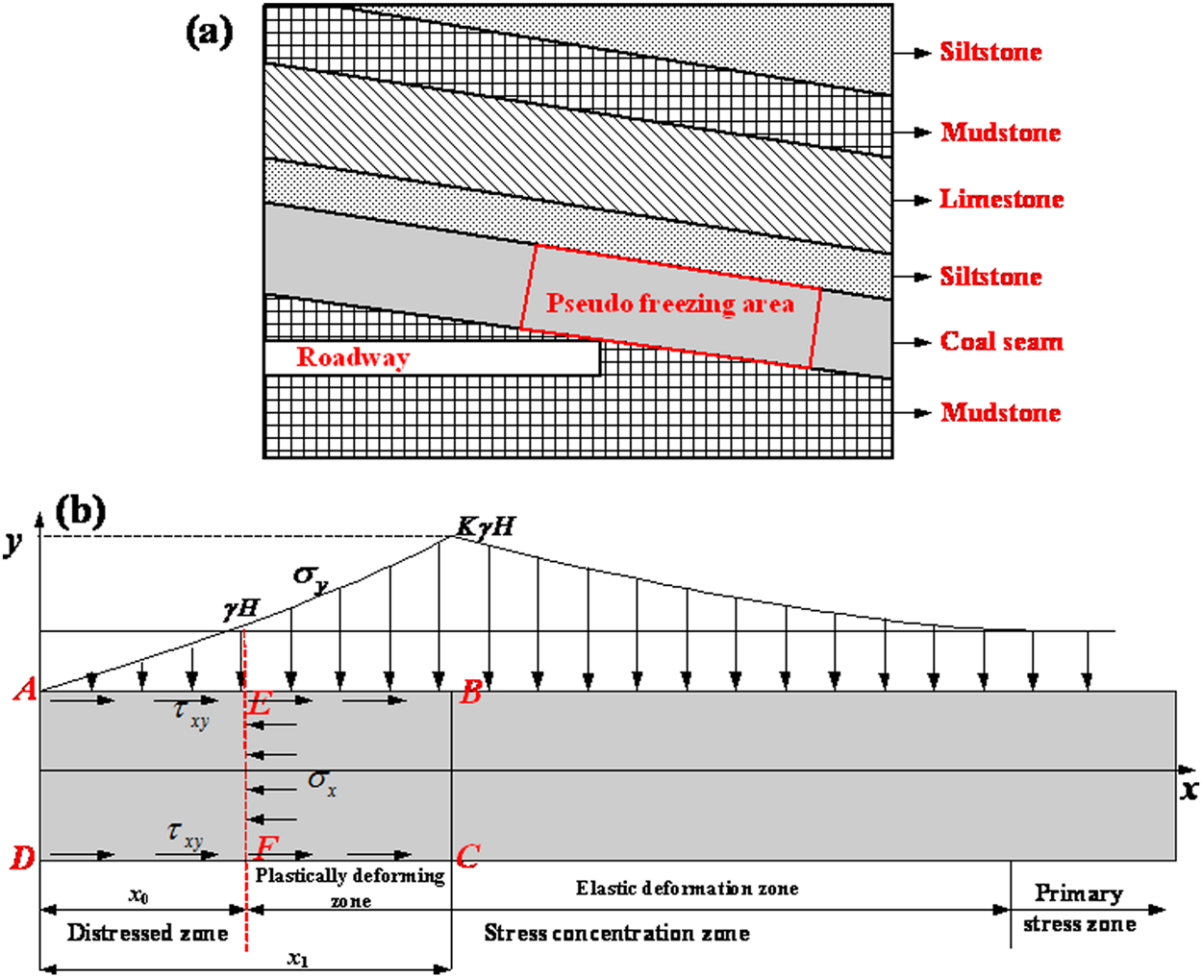
Schematics of stress distribution in a coal seam: (**a**) section of rock cross-cut coal uncovering and (**b**) stress distribution.
  To calculate the width of the distressed zone simply, it is assumed that the coal is homogeneous and isotropic in the limit stress zone and satisfies the continuous medium condition.
  The normal stress and shear stress in a coal seam increase with the distance from the roadway working face to the position of the peak stress. At the position of the peak stress, the normal stress is equal to the maximum stress in the stress concentration zone, and the horizontal stress can be expressed as the average value of the vertical stress at the interface as follows,
  1 $${[{\sigma }_{y}]}_{{x}_{1}}=K\gamma H\,{[{\sigma }_{x}]}_{{x}_{1}}=A{[{\sigma }_{x}]}_{{x}_{1}}=AK\gamma H$$
  where *σ*_*x*_ denotes the horizontal stress, *σ*_*y*_ denotes the vertical stress, *K* denotes the stress concentration factor, *γ* denotes the bulk density of the overlying strata, *H* denotes the seam depth and *A* denotes the lateral pressure coefficient.
  From limit equilibrium theory, when coal is extruded between the roof and the floor, the coal seam interface stress in the stress limit equilibrium zone should satisfy the limit equilibrium condition. Neglecting the volume force, the stress of the coal body in areas near the coal seam interface should satisfy the following differential stress equilibrium equation:
  2 $$\{\begin{array}{rcl}\frac{d{\sigma }_{x}}{dx}+\frac{d{\tau }_{xy}}{dy} & = & 0\\ \frac{d{\tau }_{xy}}{dx}+\frac{d{\sigma }_{y}}{dy} & = & 0\\ {\tau }_{xy} & = & {\sigma }_{y}\,f+c\end{array}$$
  where *τ*_*xy*_ denotes the shear stress at the floor/roof of the coal seam, *c* denotes the cohesive force, *f* denotes the interfacial friction coefficient, *f* = *tgφ*, and *φ* denotes the internal friction angle of coal.
  If the area, ABCD, of the coal body in the stress limit equilibrium zone is considered as a separator in a force analysis, the following force equilibrium condition in the *x-* direction is obtained
  3 $$2{\int }_{0}^{x}{\tau }_{xy}dx-h{\sigma }_{x}=0$$
  Equation (2) is solved as follows:
  4 $${\sigma }_{y}=\frac{c}{f}({e}^{\frac{2f}{hA}x}-1)\,{\tau }_{xy}=c{e}^{\frac{2f}{hA}x}$$
  where, *h* is the thickness of the coal seam.
  At *x* = *x*_0_, *σ*_*y*_ = *γH*, which can be substituted in to Eq. (4) to yield the width of the distressed zone (*L*_*d*_):
  5 $${L}_{d}=\frac{hA}{2f}\,\mathrm{ln}(\gamma H\frac{f}{c}+1)$$
2. Safety width of distressed zone

Experiments show that most coal destruction results from shear fracture at a certain confining pressure, even under uniaxial compression. Therefore, the coal destruction mechanism in the distressed zone is assumed to be shear fracture. Under the stress equilibrium condition, the shear strength criterion of coal in the distressed zone can be expressed as^29^,

6 $${\sigma }_{x}=\frac{{\sigma }_{t}}{{\sigma }_{c}}{\sigma }_{y}-{\sigma }_{t}$$

where *σ*_*c*_ and *σ*_*t*_ denote the uniaxial compressive strength and the tensile strength of coal, respectively.

In Fig. 1(b), the area AEFD is considered to be the object of analysis in the distressed zone, for which the horizontal resultant force is zero, that is,

7 $$h({\sigma }_{x}+p)-2{\int }_{0}^{{x}_{0}}{\tau }_{xy}dx=0$$

where *p* denotes the gas pressure acting on the coal body toward the working face.

Substituting Eqs (4) and (6) into Eq. (7) yields the following, stability condition for the coal body in the distressed zone:

8 $$N{\sigma }_{y}+p-\frac{cA}{f}({e}^{\frac{2f}{hA}{x}_{0}}-1)={\sigma }_{t}$$

where *N* = *σ*_*t*_ /*σ*_*c*_.

As $N{\sigma }_{y}+p-(cA/f)({e}^{(2f/hA){x}_{0}}-1) > {\sigma }_{t}$, coal will be destroyed in the distressed zone, which resulting in a coal and gas outburst. Therefore, to ensure coal stability in the distressed zone, the safety width of the distressed zone (*L*_*s*_) should satisfy the following condition^30,31^,

9 $${L}_{s}\ge \frac{hA}{2f}\,\mathrm{ln}[\frac{f}{cA}(N{\sigma }_{y}+p-{\sigma }_{t})+1)]$$

Equations (5) and (9) can be used to determine the danger coefficient of a coal and gas outburst at the working face of rock cross-cut coal uncovering^32^:

10 $$\omega =\frac{{L}_{s}}{{L}_{d}}=\frac{\mathrm{ln}[\frac{f}{cA}(N{\sigma }_{y}+p-{\sigma }_{t})+1)]}{\mathrm{ln}({\sigma }_{y}\frac{f}{c}+1)}$$

It can be seen from Eq. (10) that the smaller the risk coefficient of an outburst the smaller the risk of an outburst. Moreover, the risk coefficient is closely related to the coal mechanical strength.

### Interfacial elastic energy ratio between coal seam and protective rock seam

The elastic energy of a coal and rock mass is related to the stress state, the elastic modulus and Poisson’s ratio. Based on elastic mechanics, the elastic energy equation of a coal and rock mass in the distressed zone in rock cross-cut coal uncovering can be expressed as^32^

11 $$W=\frac{1}{2E}[{\sigma }_{1}^{2}+{\sigma }_{2}^{2}+{\sigma }_{3}^{2}-2\mu ({\sigma }_{1}{\sigma }_{2}+{\sigma }_{2}{\sigma }_{3}+{\sigma }_{1}{\sigma }_{3})]$$

where, *E* denotes the elastic modulus, *μ* denotes Poisson’s ratio, and *σ*_1_, *σ*_2_ and *σ*_3_ are the three principal stresses. Here, the coal and rock mass in front of the working face in rock cross-cut coal uncovering is considered to be under three-dimensional compression. In absence of a geological structure, the coal body has a low strength and can be considered to be in a hydrostatic stress state: thus, the three principal stresses are approximatiely equal, and Eq. (11) can be written as follows:

12 $$W=\frac{3{\sigma }^{2}}{2E}(1-2\mu )$$

From Eq. (12), it can be seen that the elastic energy of the coal and rock mass depends on the elastic modulus and Poisson’s ratio under certain stress conditions. Therefore, although the ultimate failure strength and elastic modulus of the protective rock seam are quite large, the elastic energy is small. By contrast, the ultimate failure strength and elastic modulus of the coal seam are low, but the elastic energy is high. The elastic energies of a coal seam and a rock seam are expressed as

13 $${W}_{C}=\frac{3{{\sigma }_{y}}^{2}}{2{E}_{C}}(1-2{\mu }_{C})$$

14 $${W}_{R}=\frac{3{{\sigma }_{y}}^{2}}{2{E}_{R}}(1-2{\mu }_{R})$$

where *W*_*C*_ and *W*_*R*_ denote the elastic energies of coal and rock, respectively; *E*_*C*_ and *E*_*R*_ denote the elastic modulus of coal and rock, respectively; and *μ*_*C*_ and *μ*_*R*_ denote Poisson’s ratio of coal and rock, respectively.

The ratio (*K*_*W*_) of the elastic energy of coal to that of rock at a coal-rock interface is expressed as

15 $${K}_{W}=\frac{{W}_{C}}{{W}_{R}}=\frac{{E}_{R}(1-2{\mu }_{C})}{{E}_{W}(1-2{\mu }_{R})}$$

During rock cross-cut coal uncovering, the elastic energy at the interface of the coal seam and the floor/roof rock can change suddenly, making the coal body extremely unstable. The larger the difference in the elastic energy between the coal seam and the floor/roof rock, the stronger the effect of the coal elastic deformation potential, which results in sudden displacement failure of the coal body and increases the chances of outburst. That is, sudden changes in mechanical and physical quantities, such as the elastic moduli of the coal and rock, will produce dynamic phenomena of coal and gas. If the gas conditions are just met at this time, a coal and gas outburst will occur. Thus, the coal strength needs to be increased, and the difference in the elastic energy between the coal and the roof/floor should be reduced.

## Mechanical Property Tests of Coal Specimens

### Production of test specimens

The experimental coal sample is obtained taken from Second-1 coal seam in the Dayugou coal mine, Gongyi, China. The general structural form of Dayugou coal mine is a monoclinic structure that runs toward N 70°W and tends toward the North East (SE). The main structural form is faults, and the folds are developed. There are twenty seven folds in the minefield. The fall of two faults is greater than 30 m. The coal is powdery anthracite with a natural moisture content of 1.6%. The coal specimens are made in strict accordance with Chinese national standards in “Methods for determining the physical and mechanical properties of coal and rock - Part 7, Part 10 and Part 11” (GB/T 23561.7–2009, GB/T 23561.10–2010). The specimen sizes for the compressive, tensile and shear tests are *Φ*50 × 100 mm, *Φ*50 × 25 mm and *Φ*50 × 20 mm, respectively. The main production processes for the coal specimens are described below.

1. The coal samples collected from the working face are crushed by a grinder, and then screened by a vibrating screen. Coal samples with diameters between 60 meshes and 80 meshes are obtained.
2. Coal fragments with diameters between 60 meshes and 80 meshes are mixed with 20% pure water, and the wet coal samples are stirred thoroughly. The self-made briquette mould is used to make the remolded coal samples. The briquette mould includes three parts, namely, the hollow cylinder, the tamping head and the retreat mould cylinder. The schematic diagram of the self-made briquette mould is shown in Fig. 2.

**Figure 2:**
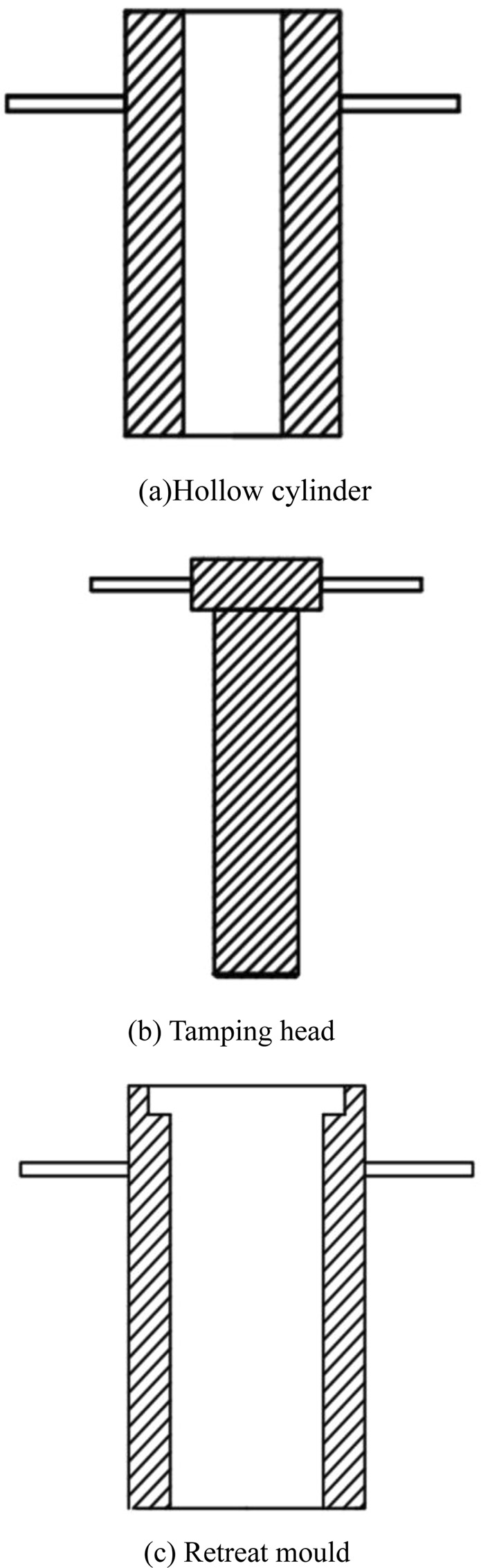
Schematics of the self-made briquette mould. (**a**) cHollow cylinder. (**b**) Tamping head. (**c**) Retreat mould.
3. The wet coal is placed in the hollow cylinder. A WES-1000B hydraulic universal testing machine is used to compress the coal samples under a load pressure of 15 MPa for 20 min. Here, a load of 15 MPa is equivalent to an *in-situ* stress at a depth of 600 m underground.
4. After the time reached 20 minutes, the coal sample is dropped out by the WES-1000B hydraulic universal testing machine. The resulting coal sample is ground until the end face flatness does not exceed 0.05 mm. Finally, the height and diameter of the coal samples are measured and recorded.
5. In the mechanical experimental test scheme including six test temperatures, four moisture contents and five parallel specimens, one hundred and forty coal specimens are used in each type of test. In addition, there are twenty coal samples for later spare.

### Moisture content control of coal specimens

Although the moisture content of coal particles is adjusted before the coal specimens are loaded, a portion of the water in the coal specimens will be squeezed out during the pressing process. To obtain coal specimens with different moisture contents, a WD-841-1 oven is used to conduct tests on the mass change of some of the coal specimens, the results of which are used to calculate the relationship between the moisture content and the drying time. The main steps are given below.

**Figure 3:**
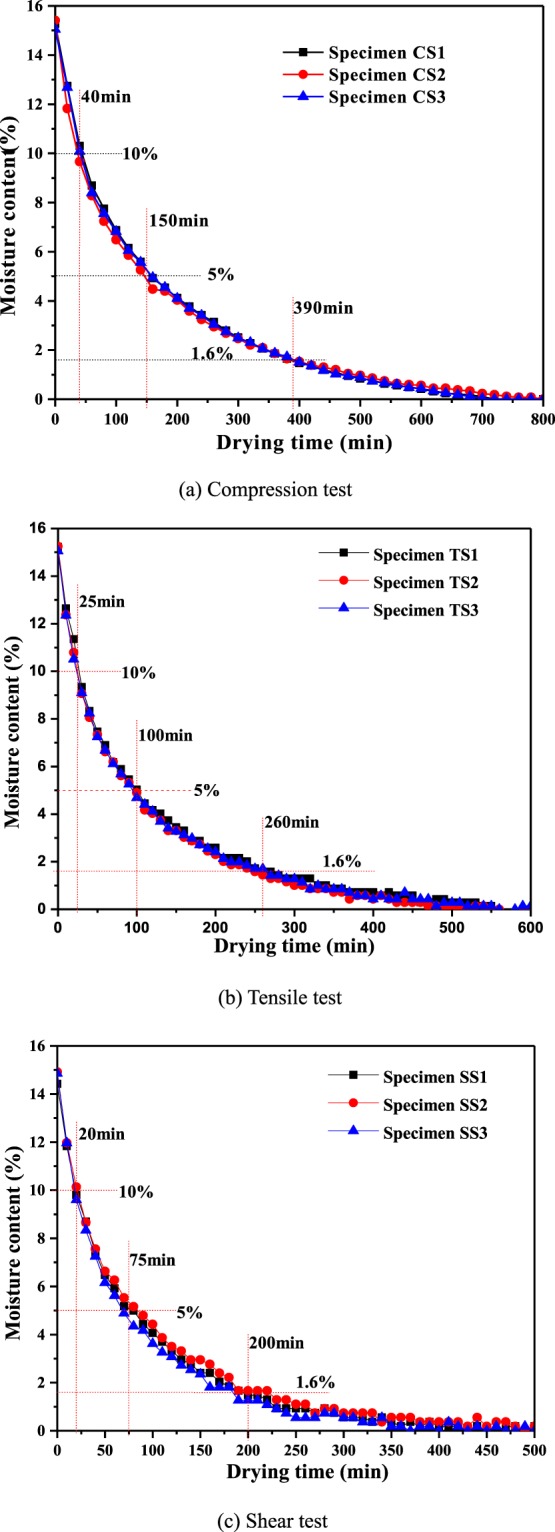
Change in moisture content of coal specimens with drying time. (**a**) Compression test. (**b**) Tensile test. (**c**) Shear test.

1. Three specimens are randomly selected for each type of specimen, and the initial mass of each specimen is weighed using a precision electronics balance.
2. To obtain the relationship between moisture content and the drying time, all of the selected coal specimens are placed in the oven, the drying process is started at 105 °C. During drying process, the coal specimens are weighed every 20 min, and the weight and corresponding time are recorded. When there is no change in the mass of a coal specimen over one hour, the moisture content is considered to be 0.
3. The change in the moisture content of the coal specimens with the drying time is shown in Fig. 3 for the uniaxial compression test, the tensile test and the shear test.

As shown in Fig. 3, coal specimens of the same type exhibited almost identical change rules for the moisture content with drying time during the drying process. Therefore, it is easy to obtain the set moisture content of the coal specimens with the set drying time based on these change rules. Using the coal specimens in the tensile test as an example (see Fig. 3(b)), for an initial moisture content of approximately 15%, when the drying time is set at 25 minutes, 100 minutes and 260 minutes, the moisture content of the coal specimens is 10%, 5% and 1.6% (natural moisture content). These test specimens with different moisture contents (1.6%, 5%, 10%, 15%) are placed in sealed bags for at least 72 h to balance their internal moisture.

### Compression, tensile and shear tests

According to the experimental objectives, the compression, tensile and shear test can be divided into three groups, namely compression test (Group I), tensile test (Group II) and outburst shear test (Group III).

#### Group I

1. The coal specimens are frozen at the setting temperature (0 °C, −10 °C, −20 °C, −30 °C and −40 °C) in a cryogenic box. To ensure that the coal specimens are completely frozen, the freezing time is maintained above 4 h.
2. The compressive strength (CS) is tested using a WES-1000B hydraulic universal testing machine, which is shown in Fig. 4. The coal samples are loaded at 0.5 mm/s.

**Figure 4:**
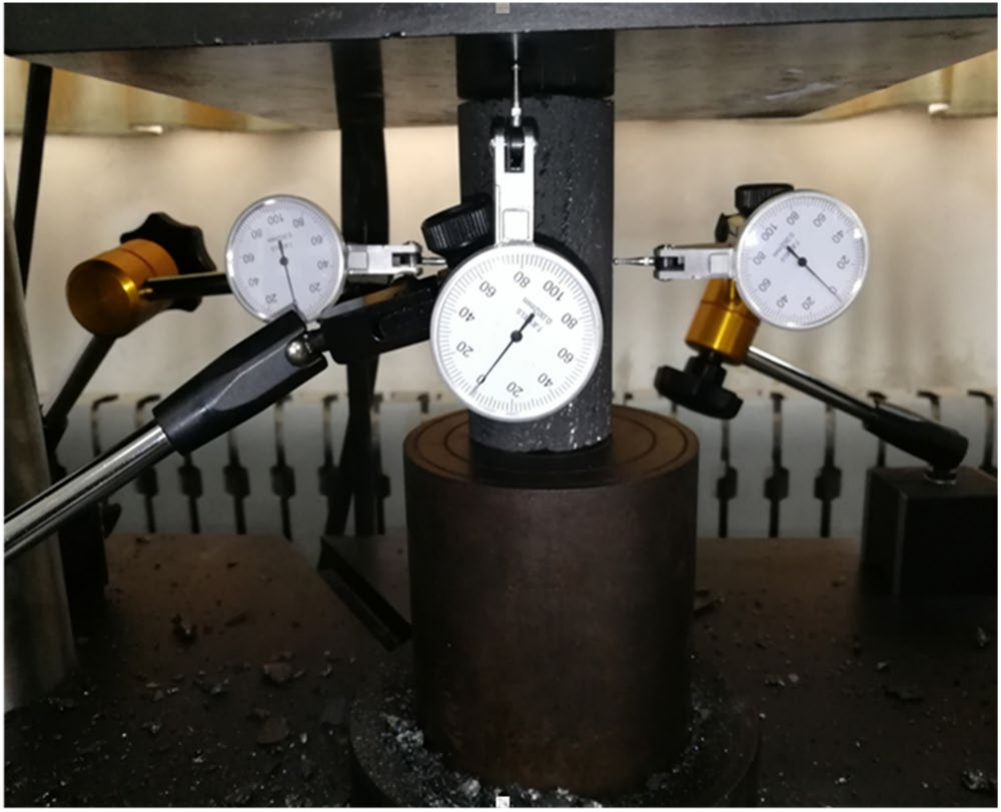
Schematics of compressive strength test.
3. When some obvious splitting failure occurs, the WES-1000B hydraulic universal testing machine is turned off. The data are saved at the same time.
4. The damaged specimens are taken out of WES-1000B hydraulic universal testing machine. Cleaning up the machine and preparing for the next group experiment.

#### Group II

1. The coal specimens are frozen at the setting temperature (0 °C, −10 °C, −20 °C, −30 °C and −40 °C) in a cryogenic box. To ensure that the coal specimens are completely frozen, the freezing time is maintained above 4 h.
2. The tensile strength (TS) was tested using a consolidometer, which is shown in Fig. 5. The specimen is placed in the center of the consolidometer. The empty bucket is hung on the hook to keep the specimen balanced.

**Figure 5:**
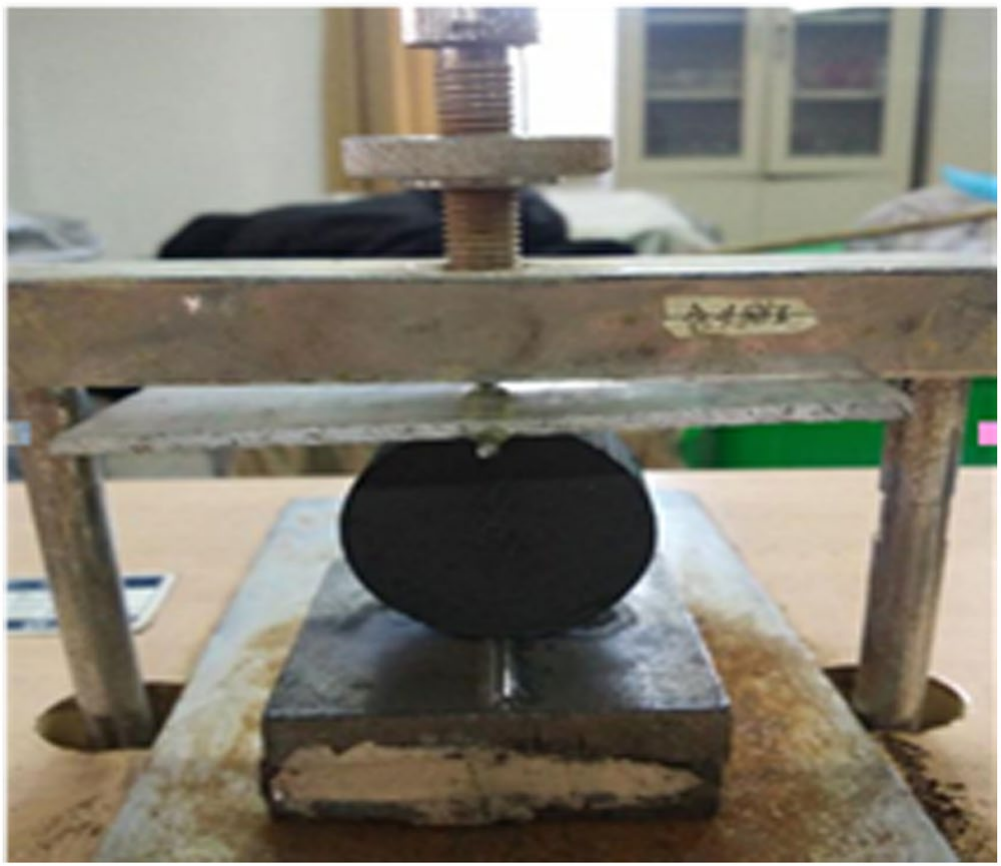
Schematics of tensile strength test.
3. Some sands are added to the bucket. The specimens are observed during the process of adding sand. If the cracks are found, the speed of adding sands should be slowed down immediately.
4. The mass of sand and the failure form of the specimen are recorded after the specimen was destroyed.

#### Group III

1. The coal specimens are frozen at the setting temperature (0 °C, −10 °C, −20 °C, −30 °C and −40 °C) in a cryogenic box. To ensure that the coal specimens are completely frozen, the freezing time is maintained above 4 h.
2. The shear strength (SS) is measured using a direct shear apparatus, which is shown in Fig. 6.

**Figure 6:**
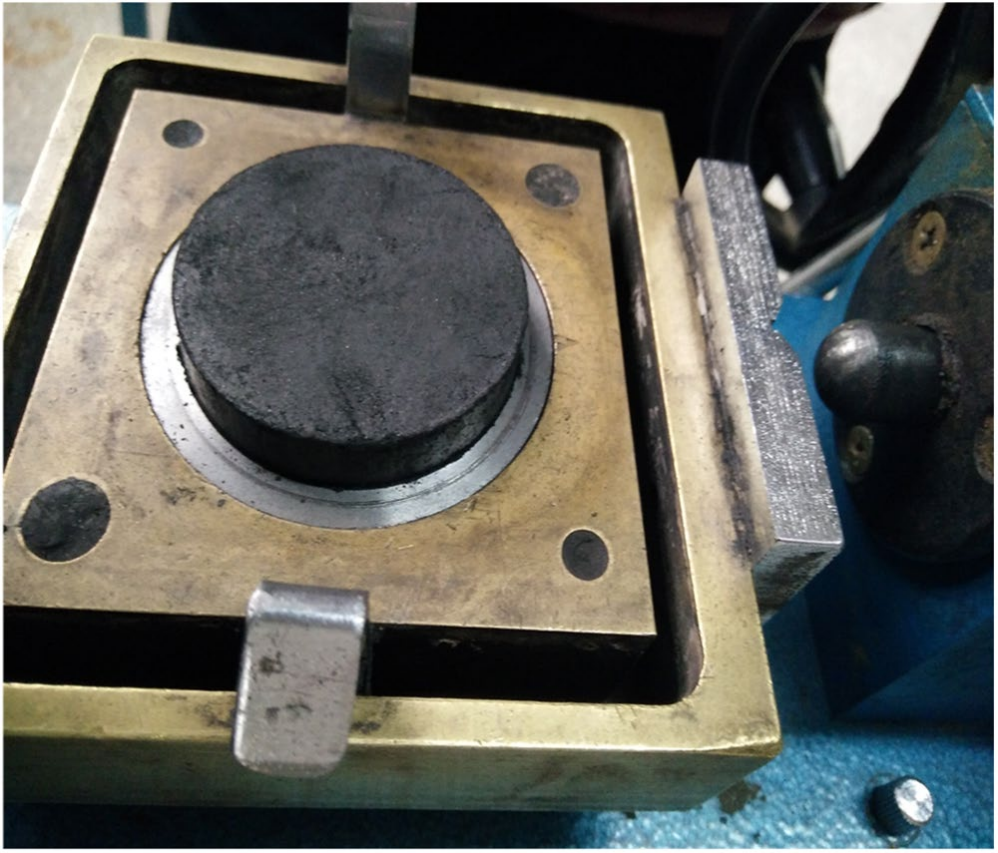
Schematics of shear strength test.
3. The frozen coal is placed in the shear box. After the sample is loading in the shear apparatus, the hand wheel is rotated slowly so that the front end of the upper box can contact the dynamometer, then the pressure cap, steel ball, pressure frame are added.
4. After the vertical load is loaded, the pin is removed immediately, and the dial gauge is set to zero. The specimen is sheared in three to five minutes until the peak value of the dynamometer appears, and the dial reading is recorded.

**Figure 7:**
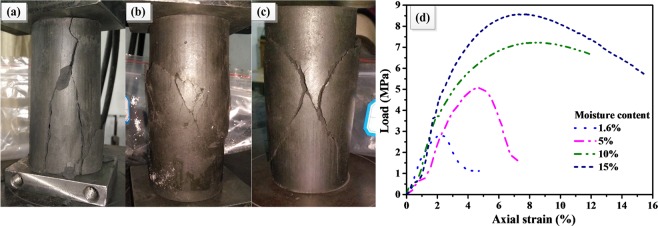
Portion of failure specimens for uniaxial compression test (at −30 °C): (**a**) 1.6%, (**b**) 10%, (**c**) and 15% (**d**) stress-strain curves.

Using the steps of the above three groups of experiments, the experiments are carried out. Portion of failure specimens for uniaxial compression tests (at −30 °C) is shown in Fig. 7. Figure 7(a–c) represent the specimen failure form at the moisture of 1.6%, 10% and 15%, respectively. Figure 7(d) represents a stress-strain curve. When moisture content of the coal specimens is low, brittle failure is clearly observed for the rock in uniaxial compression test. The main crack expands rapidly at a certain angle to the axial direction and then suddenly breaks down, where the failure basically occurs by cylindrical splitting (For example, at −30 °C, see the specimen failure form in Fig. 7(a) and the stress-strain curve in Fig. 7(d)).

However, for coal specimens with larger moisture contents (such as 10% or 15%), the coal specimens fails by plastic failure (for example, at −30 °C, see the specimen failure form in Fig. 7(b,c) and stress-strain curve in Fig. 7(d)), and bulging deformation occurs in the middle portion of the specimens during the uniaxial compression process. After the peak stress is exceeded, failure cracks are clearly visible in the specimens. However, the coal specimens remain cohesive and can withstand a certain level of compressive stress.

**Figure 8:**
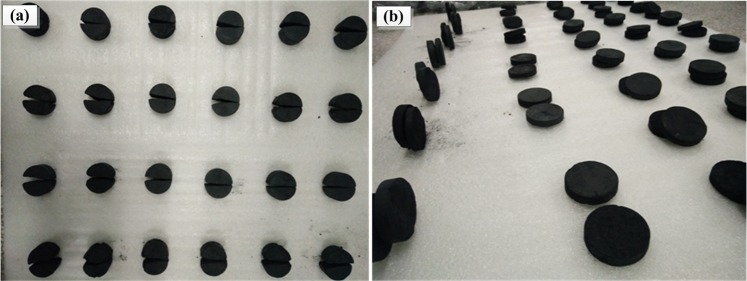
Destruction morphology: (**a**) tensile test (**b**) shear test.

In the tensile test, irrespective of the temperature of the specimens, the same failure forms are observed (as shown in Fig. 8(a)): the specimens are relatively regular and split from the middle without a significant amount of debris. The failure surface is smooth through the radial loading baseline. In the shear test, taking the failed coal specimens out of the shear box shows that the specimens have been sheared into two halves (Fig. 8(b)). The failure surface is flat, and the specimen shape is basically unchanged for different temperatures and moisture contents.

The compressive strength, tensile strength and shear strength is tested for five specimens at the same moisture content and temperature to obtain the respective mean values. The results for these mechanical parameters are calculated and shown in Table 1.

**Table 1:** Test results for mechanical parameter.

Temperature/Moisture content		Compressive strength σ_c_ (MPa)	Elasticity modulus E_C_ (MPa)	Poisson’s ratio μ_C_	Tensile strength σ_t_ (MPa)	Cohesive force c (kPa)	Interfacial friction coefficient (tgφ)
25 °C	1.6%	1.6	105.36	0.33	0.0903	154.936	0.468
	5%	1.59	78.68	0.32	0.0857	143.28	0.493
	10%	1.47	64.48	0.31	0.0957	125.852	0.493
	15%	1.34	37.64	0.32	0.0903	117.239	0.514
0 °C	1.6%	1.71	111.35	0.325	0.107	154.027	0.466
	5%	1.65	92.08	0.33	0.1003	147.943	0.445
	10%	1.49	77.45	0.32	0.0943	135.577	0.432
	15%	1.37	55.91	0.32	0.0931	120.161	0.4406
−10 °C	1.6%	1.74	115.67	0.33	0.113	154.933	0.482
	5%	2.34	120.5	0.34	0.15	155.393	0.424
	10%	2.89	139.22	0.34	0.2097	145.816	0.402
	15%	3.67	164.08	0.35	0.2592	135.85	0.45
−20 °C	1.6%	2.27	130.85	0.32	0.1198	158.781	0.461
	5%	3.84	167.87	0.35	0.1952	164.465	0.437
	10%	5.63	223.9	0.36	0.3011	169.005	0.442
	15%	7.56	294.7	0.38	0.3635	173.073	0.45
−30 °C	1.6%	2.78	153.58	0.33	0.1332	164.79	0.514
	5%	5.2	209	0.37	0.267	175.17	0.46
	10%	7.37	279.93	0.39	0.4187	199.3	0.479
	15%	9.56	374.5	0.42	0.4953	234.49	0.454
−40 °C	1.6%	3.87	179.25	0.34	0.1599	176.326	0.5
	5%	7.49	285.63	0.39	0.354	189.289	0.503
	10%	10.38	356.3	0.42	0.5346	242.851	0.458
	15%	12.46	455.07	0.46	0.6615	335.6	0.471

## Mechanical Analysis of Safety Performance in RCCCU

### Strength enhancement of frozen coal

**Figure 9:**
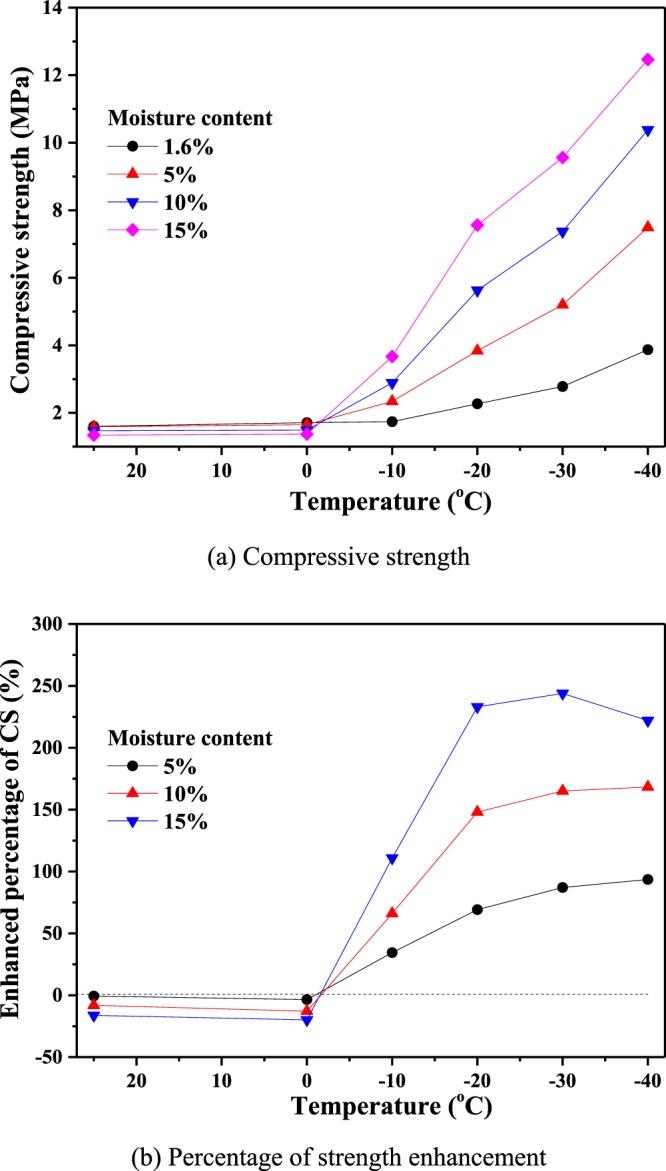
Compressive strength of coal specimens at different temperatures. (**a**) Compressive strength. (**b**) Percentage of strength enhancement.

Using the results in Table 1, the uniaxial compressive strengths for the coal specimens with different moisture contents and at different freezing temperatures are shown in Fig. 9. From Fig. 9(a), it can be seen that the temperature has little effect on the compressive strength above 0 °C, and the larger the moisture content, the smaller the compressive strength. However, for temperatures below 0 °C, the compressive strength exhibits minimal variation with the temperature. By comparison, for coal with a natural moisture content (1.6%) at the same freezing temperature, the larger the moisture content, the more significant the change in the compressive strength (as shown in Fig. 9(b)). For example, at a freezing temperature of −20 °C, the compressive strength is enhanced by 69.16%, 148.02% and 233.04% for moisture contents of 5%, 10% and 15%, respectively. This result is obtained because the water molecules in the cracks and pores of the coal body effectively cement the coal matrix during freezing. The larger the moisture content, the stronger the cementation effect. The coal-water body is frozen into a whole, which clearly enhances the coal compressive strength.

**Figure 10:**
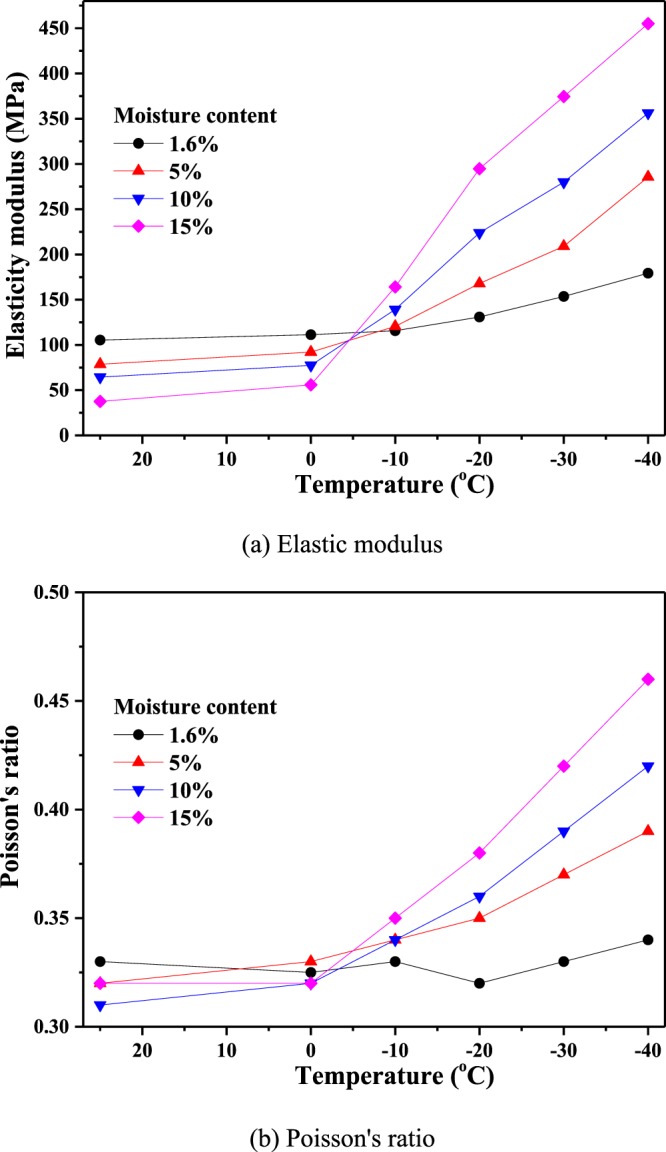
Elasticity modulus and Poisson’s ratio of coal specimens at different temperatures. **(a**) Elastic modulus, (**b**) Poisson’s ratio.

As shown in Fig. 10, the elastic modulus and Poisson’s ratio of frozen coal with different moisture contents exhibit similar change trends as the compressive strength. That is, the lower the freezing temperature, the larger the elastic modulus and Poisson’s ratio. For example, when the freezing temperature is −20 °C, and the moisture contents are 5%, 10% and 15%, the elastic modulus of coal is 28.29%, 71.11% and 125.22%, respectively, larger than coal with a natural water content (1.6%), and the Poisson’s ratio increases by 9.38%, 12.5% and 18.75%, respectively.

**Figure 11:**
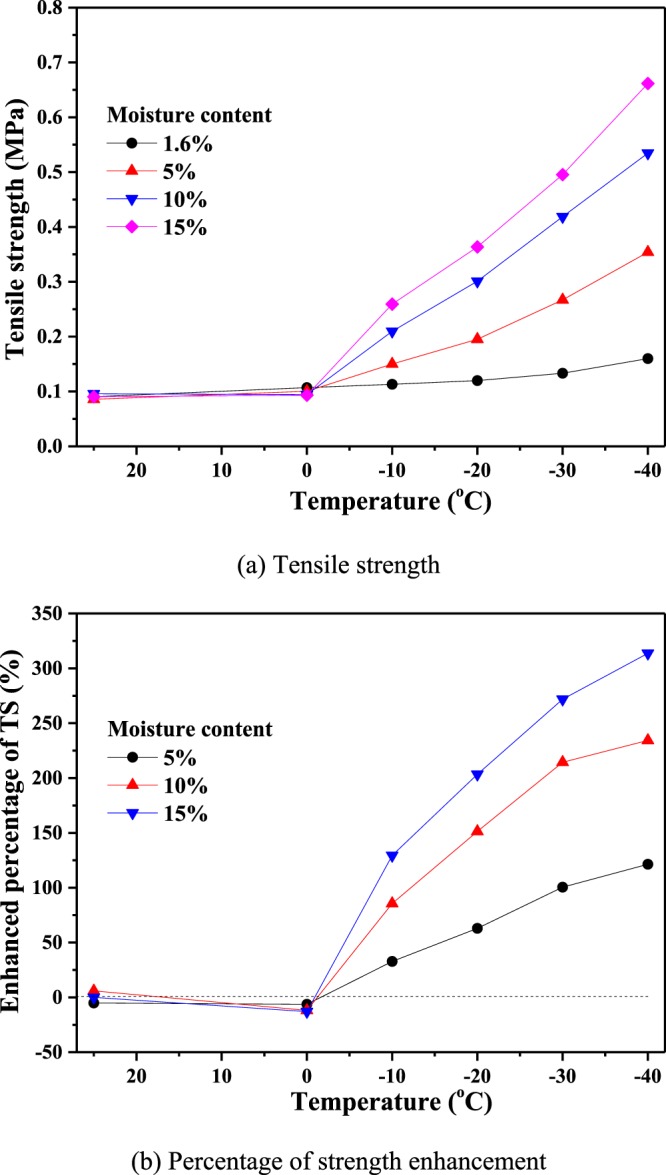
Tensile strength of coal specimens at different temperatures. (**a**) Tensile strength. (**b**) Percentage of strength enhancement.

As shown in Fig. 11, below 0 °C, the tensile strength of coal increases linearly as the temperature decrease. At same freezing temperature, the tensile strength increases with moisture content. For example, when the freezing temperature is −20 °C, and the moisture content is 5%, 10% and 15%, the tensile strength is 62.94%, 151.34% and 203.42%, respectively, larger than coal with a natural water content (1.6%).

**Figure 12:**
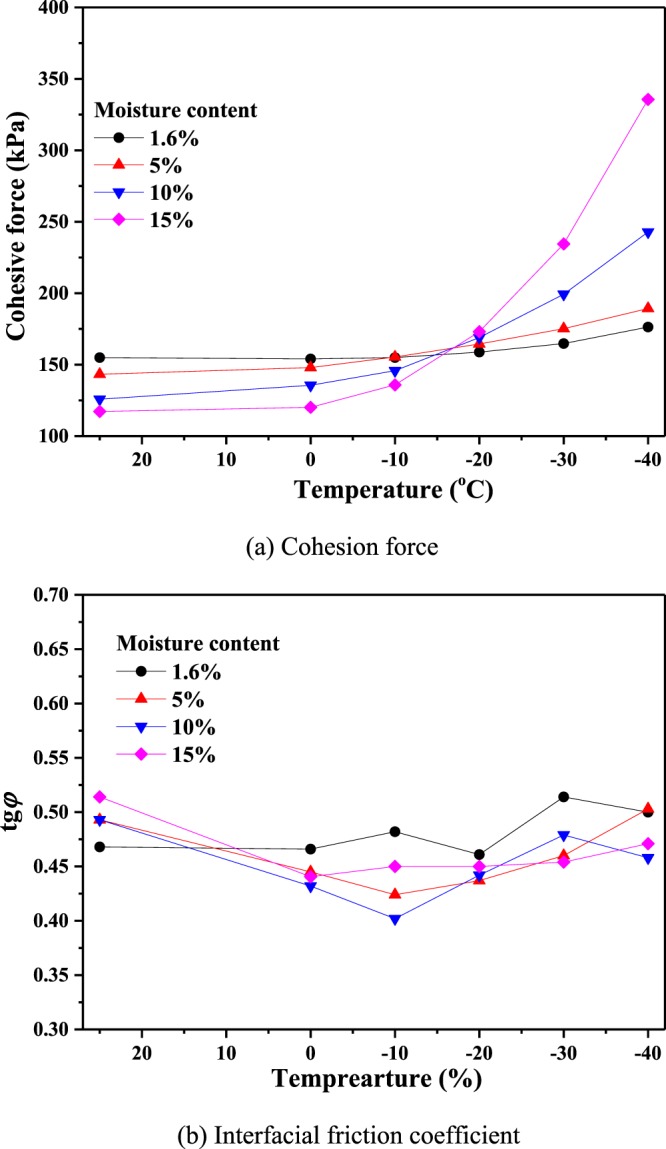
Shear parameters of coal specimens at different temperatures. (**a**) Cohesion force. (**b**) Interfacial friction coefficient.

In Fig. 12, the cohesion force (*c*) and the interfacial friction coefficient (*tgφ*) are used to measure the shear strength of coal. From Fig. 12(a), it can be seen that the temperature (above 0 °C) has little effect on the cohesion force for the same moisture content, and the cohesion force decreases as the water content increases. However, for temperatures below 0 °C, the cohesion force increases gradually, especially for large moisture contents. However, the interfacial friction coefficient, which is proportional to the interfacial friction angle (*φ*), does not change significantly with the temperature (Fig. 12(b)).

In general, for coal specimen with a large moisture content, the mechanical properties are significantly improved under freezing conditions, and decreasing the temperature rapidly enhances the compressive strength, tensile strength and shear strength of coal. In rock cross-cut coal uncovering, water is injected into the coal seam to increase its moisture content, and after a period of time, the refrigerant (liquid nitrogen) injected into the coal seam. In a freezing environment, water and coal (including adsorbed gas) are frozen into a three-phase body, which improves the mechanical properties of coal body remarkably. Then, the former outburst coal seam becomes a continuous and uniform frozen wall with a sufficiently high strength that considerable energy is required to break and throw coal during the outburst process. Therefore the coal seam resistance for outburst increases and provides a favourable guarantee for the safe production of coal and gas outburst prevention.

### Reduction of coefficient of outburst hazard in distressed zone

Here, the rock cross-cut coal uncovering at the Second-1 coal seam in Dayugou coal mine, Gongyi, China, is used as an example. The thickness of the coal seam approximately 4.62 m, and the seam depth is approximately 600 m. The relationship between the rock bulk density (*γ*_*i*_) and the burial depth (*H*_*i*_) of the overlying strata can be used to calculate the *in-situ* stress on the coal seam (${\sigma }_{y}=\sum {\gamma }_{i}{H}_{i}$) as approximately 15 MPa, and the lateral pressure coefficient is *A* = *μ*/(1 − *μ*), where *μ* denotes Poisson’s ratio.

**Figure 13:**
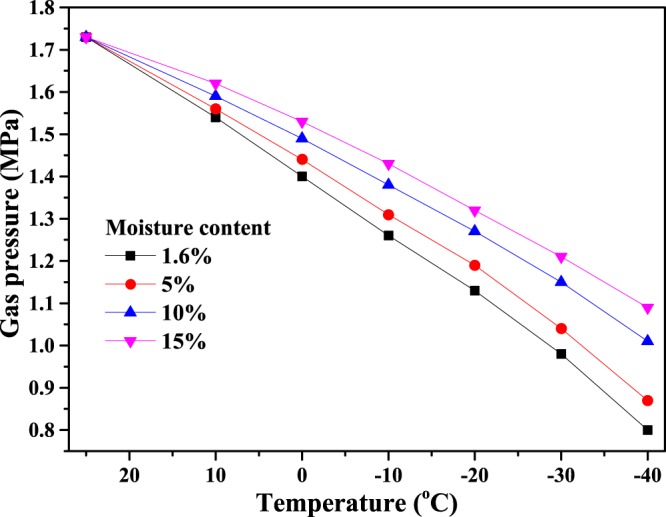
Gas equilibrium pressure for coal in a sealed container at different moisture contents and temperatures.

The change in gas pressure for coal is also tested during the cooling process. Under the same initial temperature (25 °C) and gas adsorption equilibrium pressure (1.73 MPa, the initial gas pressure in the coal seam), the pressures of gas re-adsorption equilibrium in coal with different moisture contents are tested at different temperatures in a sealed container, as shown in Fig. 13. It can be seen that from as the temperature decreases, the gas pressure in the sealed container decreases rapidly. At the same temperature, the smaller the coal moisture content, the lower the gas pressure, which indicates that lowering the temperature not only enhances gas adsorption ability in coal (i.e., gas adsorption capacity increases) but also decreases the free gas pressure^33–35^. This result is obtained because the water molecules occupy some of the adsorption sites in the coal body, and the larger the moisture content, the fewer the number of residual adsorption sites. Therefore, as the moisture content increases, the gas adsorption capacity in coal decreases and there is a relatively larger pressure from the increased amount of free gas.

**Figure 14:**
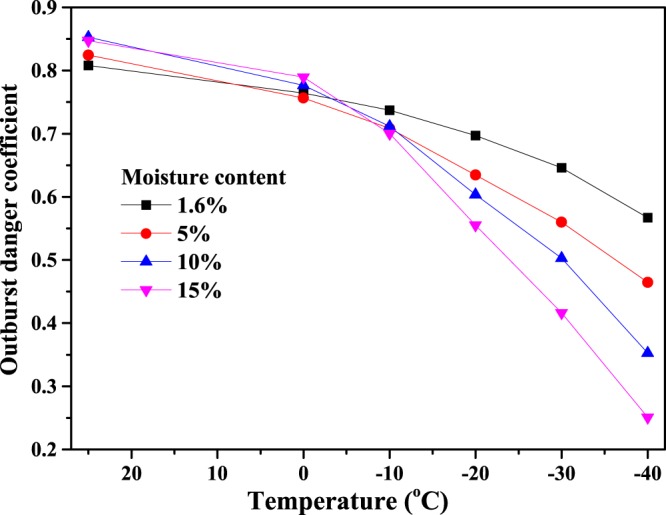
Coefficient of outburst hazard in rock cross-cut coal uncovering at different moisture contents and temperatures.

The mechanical parameters in Table 1 and the gas pressure in Fig. 13 are substituted into Eq. (8) to calculate, the danger coefficients of coal and gas outburst in rock cross-cut coal uncovering under different moisture contents and temperatures, as shown in Fig. 14. It can be seen from Fig. 14 that the coefficient of outburst hazard is larger above 0 °C in rock cross-cut coal uncovering. However, below 0 °C, the coefficient of outburst hazard decreases gradually as the temperature decreases at the same moisture content: the larger the coal moisture content, the smaller the coefficient of outburst hazard in freezing temperatures. For example, when the moisture content is 5%, 10% and 15% at −20 °C, the coefficient of outburst hazard is 0.63, 0.60 and 0.56, respectively. When the temperature drops to −40 °C, the coefficient of outburst hazard decreases to 0.46, 0.35 and 0.25, respectively. Therefore, the coefficient of outburst hazard is effectively reduced by freezing the coal seam after injecting water, which greatly reduces the danger of coal and gas outburst in rock cross-cut coal uncovering.

### Reduction of interfacial elastic energy ratio between coal seam and protective rock seam

Usually, the elastic modulus of floor/roof rocks is one order of magnitude larger than that of the coal seam; thus, the elastic energy of the coal seam is much larger than that of rock. In engineering applications, appropriate measures should be taken to weaken sudden changes in the elastic energy at the coal- rock interface in front of the roadway to reduce the risk of outburst in rock cross-cut coal uncovering.

**Figure 15:**
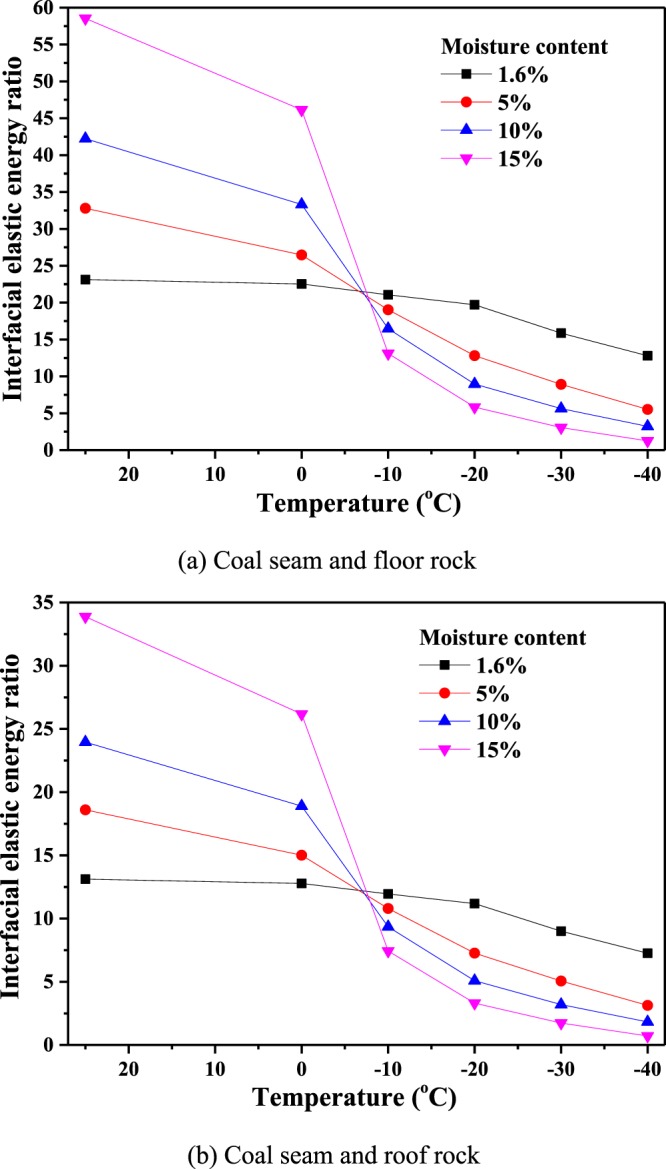
Interfacial elastic energy ratio for coal at different moisture contents and temperatures. (**a**) Coal seam and floor rock. (**b**) Coal seam and roof rock.

The floor and roof rock of the Second-1 coal seam in the Dayugou coal mine, Gongyi, China, are made of mudstone and siltstone, respectively. The elastic modulus and Poisson’s ratio of these materials are shown in Table 2. Herein, neglecting the temperature change in the rock from the frozen coal seam, the parameters of floor/roof rock are considered to be constants. Therefore, the values of the elastic modulus and Poisson’s ratio of coal in Table 1 are substituted into Eq. (13) to calculate the interfacial elastic energy ratio of coal to rock, as shown in Fig. 15. It can be seen that the interfacial elastic energy ratio decreases with decreasing temperature: especially below 0 °C, the ratio drops sharply and then decreases gradually; the larger the moisture content, the more significant the change in the ratio. For example, for a moisture content of 10% at temperatures of 25 °C, −20 °C and −30 °C, the interfacial elastic energy ratio of the coal seam and floor rock is 42.24, 8.96 and 5.63, respectively. The interfacial elastic energy ratio of the coal seam to the roof rock is 23.96, 5.08 and 3.19, respectively. Before the coal seam is frozen, the elastic energy of the coal seam is dozens of times greater than that of the floor/roof rock; however, after the coal seam is frozen, the elastic energy of the coal seam is only several times larger than that of the floor/roof rock and approaches close to the elastic energy of rock at sufficiently low temperatures. These results indicate that after the coal seam is injected with water, and then liquid nitrogen, the elastic energy of the coal seam is reduced significantly, which can weaken a sudden change in the interfacial elastic energy between the coal seam and the floor/roof rock and restrain a coal and gas outburst in rock cross-cut coal uncovering.

**Table 2:** Floor/roof rock parameters.

Rock	Lithology	elastic modulus (GPa)	Poisson’s ratio
Floor	Mudstone	2.15	0.35
Roof	Siltstone	1.87	0.27

## Discussion of Engineering Application

Coal and gas outburst in rock cross-cut coal uncovering is a serious disaster, which is a difficult problem in coal mining. The mechanism of coal and gas outburst includes three aspects, namely, the gas pressure, the *in-situ* stress and the mechanical properties of coal. The new method and new technology for preventing and controlling coal and gas outburst in rock cross-cut coal uncovering are proposed in this paper. According to the results of test and analysis, the coal strength of the frozen coal seam is greatly improved, and the difference of elastic energy between the roof of coal seam and floor is greatly reduced. The methane adsorption quantity increases with decreasing temperature. The free gas quantity decreases with temperature. Therefore, the gas pressure decreases with temperature. The new method can effectively reduce the gas pressure and weaken the gas outburst expansion energy.

Gas pressure is positively correlated with the intensity of coal and outburst. The mechanical properties of coals are negatively correlated with the intensity of coal and outburst^36–40^. Therefore, the new method plays an important role in the prevention and control of coal and gas outburst. However, application of this technology in engineering practice needs further study. The liquid nitrogen needs to be injected by borehole. The coal seam is heterogeneous. The physical parameters of the coal seam are different such as permeability, thermal conductivity and porosity, which may cause different freezing areas around the borehole. Therefore, the aging radius of freezing area needs to be grasped, which can provide guidance for the arrangement distance of borehole. The quantity of liquid nitrogen injected and nitrogen injection cycle also need to be mastered, which can avoid wasting resources. Investigation of the mechanical properties of a frozen coal can present a theoretical foundation for the application of freeing method for preventing and controlling coal and gas outburst in rock cross-cut coal uncovering.

## Conclusions

For practical engineering problems involving the high danger of coal and gas outbursts in rock cross-cut coal uncovering, a freezing method for rock cross-cut coal uncovering is proposed to prevent coal and gas outbursts. The mechanical properties (the compressive strength, the tensile strength and the shear strength) of coal specimens are tested and analysed at different water contents and freezing temperatures. These mechanical parameters are used to theoretically calculate and analyse the coefficient of outburst hazard in the distressed zone and the interfacial elastic energy ratio of coal seam and floor/roof rock. The results are summarized below,

1. The mechanical properties of coal are greatly improved after the wet coal is frozen. Below 0 °C, the larger the coal moisture content, the more significant the enhancement of the mechanical parameters: the compressive strength and tensile strength, in particular, are several times larger than before freezing.
2. The coefficient of outburst hazard has been effectively reduced in the distressed zone. Before freezing, the coefficient of outburst hazard in rock cross-cut coal uncovering is more than 0.8. However, after freezing, the coefficient of outburst hazard drops to be below 0.5 and even to below 0.3 at −40 °C. Moreover, at the same freezing temperature, the larger the moisture content of coal, the smaller the coefficient of outburst hazard.
3. Interfacial elastic energy ratio of the coal seam and the floor/roof rock is greatly reduced. The interfacial elastic energy ratio decreases sharply at freezing temperatures, which reduces the difference in the elastic energy between the coal seam and the floor/roof rock.

This study will provide a theoretical basis for the development of new technology for preventing and controlling coal and gas outburst in rock cross-cut coal uncovering.
